# Supplementary material for: Unveiling the Exquisite Microstructural Details in Zebrafish Brain Non-Invasively Using Magnetic Resonance Imaging at 28.2 T
Source: Molecules. 2024 Sep 29;29(19):4637. doi: 10.3390/molecules29194637 (PMC11477492; doi:10.3390/molecules29194637)
Supplement: Supplementary file 1 [file molecules-29-04637-s001.zip › molecules-3202824-supplementary.pdf]

Unveiling the Exquisite Microstructural Details in Zebrafish Brain Non-invasively using MRI at 28.2 T

Rico Singer[1], Ina Oganezova[1], Wanbin Hu[2], Yi Ding[2], Antonios Papaioannou[3], Huub J.M. de Groot[1], Herman P. Spaink[2], and A Alia[1,4*]

[1] Leiden Institute of Chemistry, Leiden University, Einsteinweg 55, 2301 RA Leiden, The Netherlands, a.alia@chem.leidenuniv.nl
[2] Institute of Biology, Leiden University, Einsteinweg 55, 2301 RA Leiden, The Netherlands, h.p.spaink@biology.leidenuniv.nl
[3] Bruker BioSpin GmbH, NMR Microscopy, Rheinstetten, Germany, antonios.papaioannou@bruker.com
[4] Institut für Medizinische Physik und Biophysik, Universität Leipzig, Härtelstr. 16-18, D-04107 Leipzig,
Germany, Alia.AliaMatysik@medizin.uni-leipzig.de
* Correspondence a.alia@chem.leidenuniv.nl; Tel.: (0341) 97-15707

Supporting Information Available

Figure S1. Superior identification of brain structures based on diffusion weighted imaging (DWI) contrast as compared to RARE image contrast.

Figure S2. Response function estimation from DTI dataset of adult zebrafish brain.

Figure S3. 3D DTI results of adult zebrafish brain, showing coronal, sagittal, and axial slices, acquired at 28.2 T.

Figure S4. Schematic overview of experimental setup, for performing magnetic resonance imaging of zebrafish brain at 28.2 T (1.2 GHz)

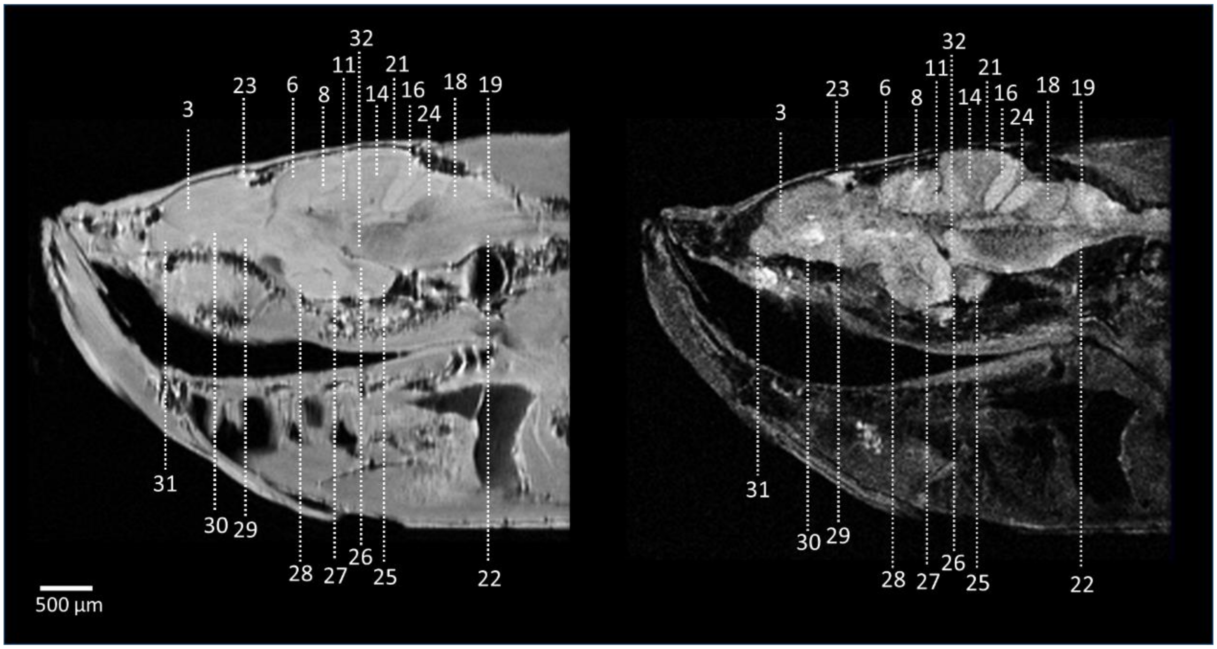

Figure S1. **Superior identification of brain structures based on diffusion weighted imaging (DWI) contrast as compared to RARE image contrast.** A representative sagittal slice of RARE image taken in the head of a zebrafish acquired at 28.2 T. RARE acquisition details: TR 3000 ms, TE 5.6 ms, 16 averages, resolution 23 µm × 23 µm × 200 µm, RARE factor 4. SNR maps generated from RARE images (right column); [B] A representative sagittal slice of DWI measurement taken in the head of a zebrafish at a b-values of 1000 s/mm2. Acquisition details: TR 1000 ms, TE 20.2 ms, 4 averages, resolution 23 µm × 23 µm × 200 µm. Structure numbering (kept same as in Fig. 2): 3 – Medial zone of dorsal telencephalon; 4 – Posterior zone of dorsal telencephalon area; 5 – Dorsal habenular nucleus; 6 – Optic tectum; 7 – Tectal ventricle; 8 – Longitudinal torus; 9 – Periventricular gray zone of optic tectum; 10 – Ventrolateral nucleus of semicircular torus; 11 – Medial division of valvula cerebelli, molecular level; 12 - Medial division of valvula cerebelli, granular layer; 13 – Granular eminence; 14 – Cerebellar corpus, granular layer; 15 – Rhombencephalic ventricle; 16 – Caudal lobe of cerebellum; 17 – Medial octavolateralis nucleus; 18 – Facial lobe; 19 – Vagal lobe; 20 – Medial funicular nucleus; 21 – Cereberal corpus, molecular layer; 22 – Medial longitudinal fascicle; 23 – Dorsal sac, 24 – Cerebellar crest; 25 – Diffuse nucleus of the inferior lobe; 26 – Mammillary body; 27 – Caudal zone of periventricular hypothalamus; 28 – Ventral zone of periventricular hypothalamus; 29 – Parvocellular preoptic nucleus, anterior part; 30 – Ventral nucleus of ventral telencephalon area; 31 – Olfactory bulb; 32 – Interpeduncular nucleus.

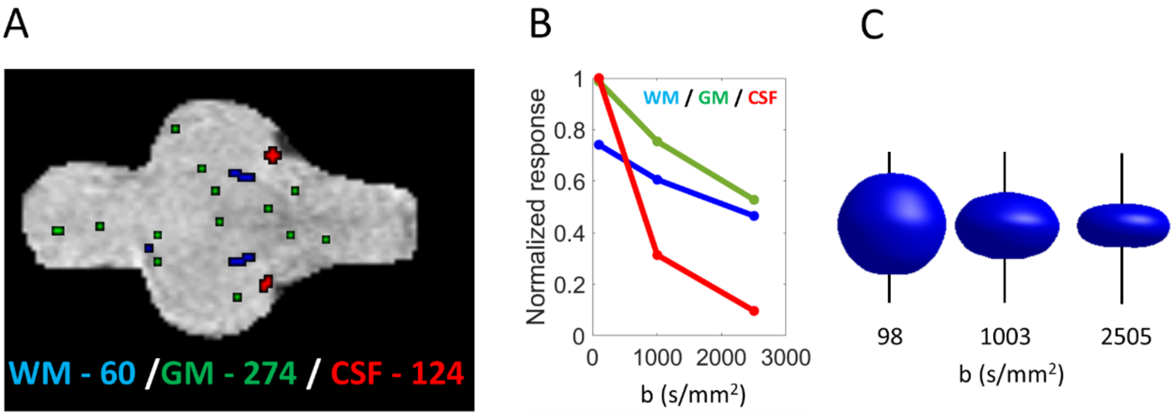

Figure S2. **Response function estimation from DTI dataset of adult zebrafish brain. [A]** Voxels used for the estimation of response function; WM (blue), GM (green), and CSF (red). The total number of voxels selected for the response function estimation was 60 (WM), 274 (GM), and 124 (CSF). **[B]** Average normalized tissue response for WM, GM, and CSD as a function of *b*-values, **[C]** average angular WM response at applied b-values. Pure WM (blue), GM (green), and CSF (red) voxels from 3D DTI zebrafish brain data were automatically selected and used for the response function estimation. Fig. S2(B) shows the estimated normalized average tissue responses of WM, GM, and CSF as a function of applied b-values. At b = 0 s/mm^2, CSF and GM show a similar response, while WM response is approximately 20% lower. Towards b = 1000 and 2500 s/mm^2, CSF shows a strong signal decline originating from the diffusivity of its free water content. Furthermore, the response of GM is higher than WM at b = 1000 and 2500 s/mm^2. However, towards higher *b*-values, this difference becomes smaller. These observations are consistent with WM, GM, and CSF response functions estimated by msmt-CSD for human brain data by Jeurissen et al (Jeurissen et al., 2014) [1]. [C] shows the average angular response for WM as a function of the applied *b*-values. At increasing *b*-values, the amplitude of the WM response decreases while the signal response is increasingly anisotropic. These results are also consistent with previous reports of WM response functions in the human brain (Jeurissen et al., 2014) [1] and murine brain data (Jillings et al., 2020) [2]. Utilizing identical processing previously applied on human and murine brains, we successfully applied msmt-CSD on zebrafish DTI data. No additional adjustments for data processing were required, although for 2D DTI data, the percentage of WM voxels for response function estimation was elevated to 10% due to the low number of total available voxels.

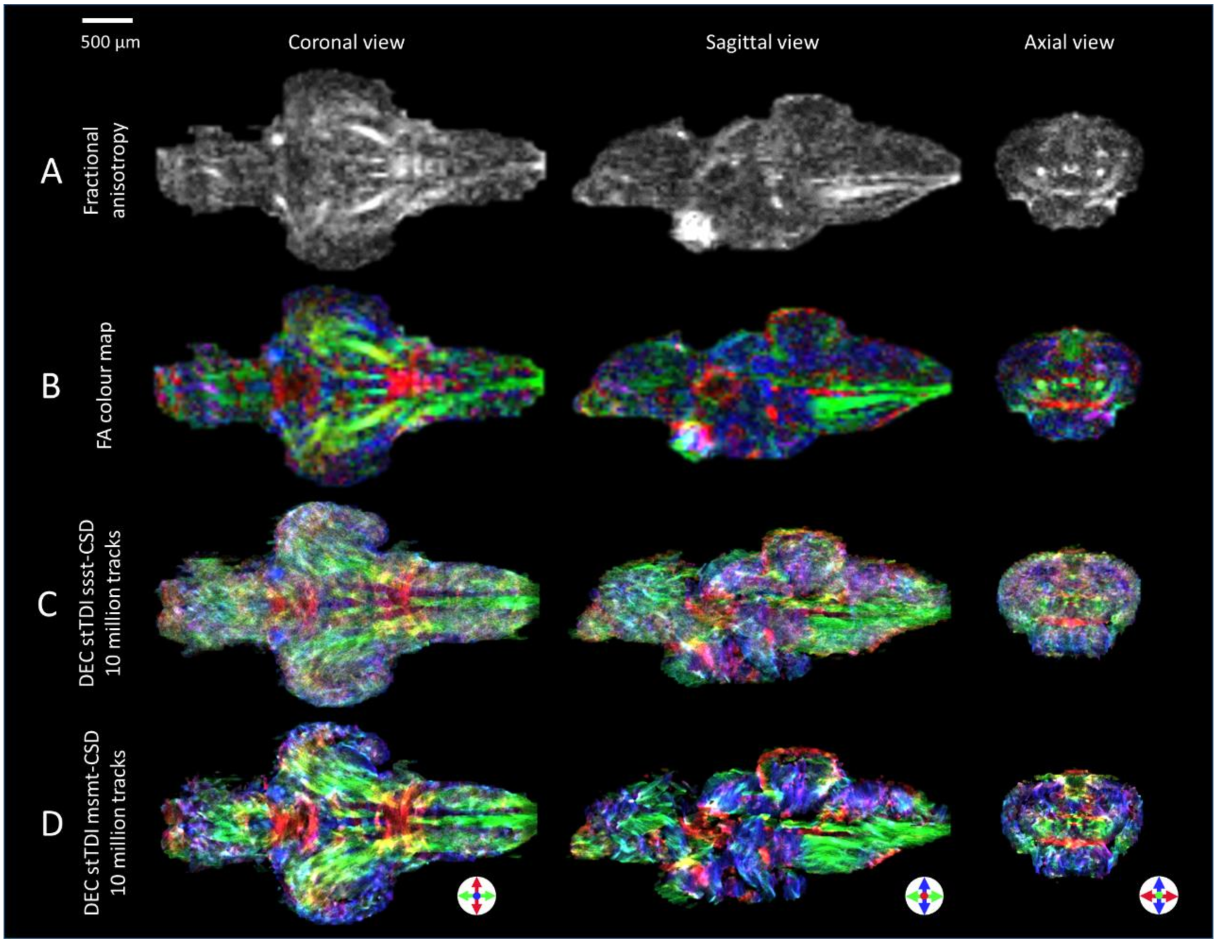

Figure S3. 3D DTI results of adult zebrafish brain, showing coronal, sagittal, and axial slices, acquired at 28.2 T. Diffusion tensors were used to calculate the **[A]** fractional anisotropy (*FA*) and **[B]** *FA* colour map. Short track (st) tractography with constrained spherical deconvolution (CSD) was applied, comparing two different algorithms for the estimation of the response function. **[C]** Super-resolution DEC stTDI maps calculated from single-shell single-tissue (ssst) CSD using the tournier algorithm [3] to estimate the response function. [D] Super-resolution DEC stTDI map calculated from white-matter response function which was estimated by multi shell multi tissue (msmt) CSD using the dHollander algorithm [4]. Tractography performed by generating ten million streamlines, with a minimal length of twice the voxel size (70 µm) and a maximum length of ten times the voxel size (350 µm). DEC is used to indicate orientation; green – rostral/caudal, blue – dorsal/ventral, and red – medial/lateral. Acquisition details: *TR* 2000 ms, *TE* 9 ms, 4 averages, isotropic resolution 35 µm, effective *b*-value range 100, 1000, 2500 s/mm^2, with 4, 12 and 24 directions respectively.

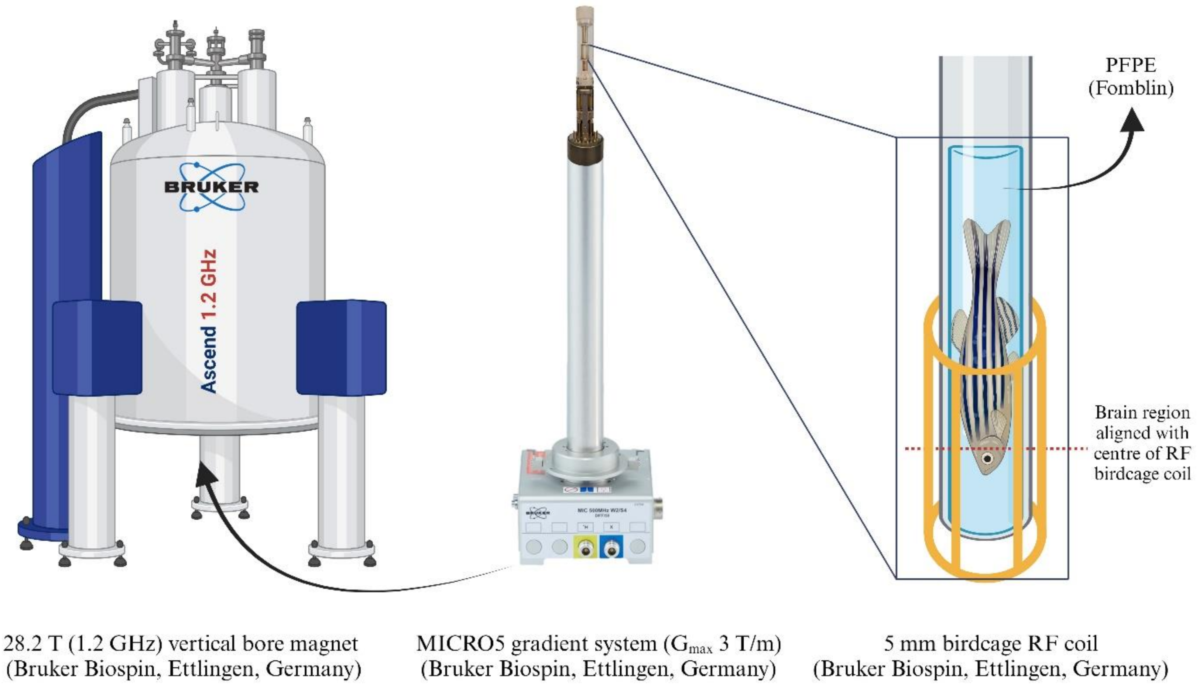

Figure S4. Schematic overview of experimental setup, for performing magnetic resonance imaging of zebrafish brain at 28.2 T (1.2 GHz). The MRI experiments were performed using vertical bore magnets (Bruker Biospin, Ettlingen, Germany), equipped with a MICRO 5 gradient system capable of a maximum gradient strength of 3 T/m and a 5 mm birdcage RF coil. Intact zebrafish were placed in 5 mm NMR tubes and immersed in perfluoropolyether (Fomblin Y, Solvay Solexis S.P.A.) to prevent dehydration and suppress background signals. To maximize signal intensity from the zebrafish brain, the brain region was precisely aligned with the centre of the birdcage RF coil.

References

1. Jeurissen B, Tournier JD, Dhollander T, Connelly A, Sijbers J (2014) Multi-tissue constrained spherical deconvolution for improved analysis of multi-shell diffusion MRI data. *Neuroimage* **103**, 411-426.
2. Jillings S, Morez J, Vidas-Guscic N, Van Audekerke J, Wuyts F, Verhoye M, Sijbers J, Jeurissen B (2020) Multi-tissue constrained spherical deconvolution in a murine brain. *Proc. Intl. Soc. Mag. Reson. Med.* **28**.
3. Tournier JD, Calamante F, Connelly A (2013) Determination of the appropriate b value and number of gradient directions for high-angular-resolution diffusion-weighted imaging. *NMR Biomed* **26**, 1775-1786.
4. Dhollander T, Raffelt D, Connelly A (2016) *Unsupervised 3-tissue response function estimation from single-shell or multi-shell diffusion MR data without a co-registered T1 image.*
